# Endoplasmic Reticulum Quality Control Is Involved in the Mechanism of Endoglin-Mediated Hereditary Haemorrhagic Telangiectasia

**DOI:** 10.1371/journal.pone.0026206

**Published:** 2011-10-14

**Authors:** Bassam R. Ali, Imen Ben-Rebeh, Anne John, Nadia A. Akawi, Reham M. Milhem, Nouf A. Al-Shehhi, Mouza M. Al-Ameri, Shamma A. Al-Shamisi, Lihadh Al-Gazali

**Affiliations:** 1 Department of Pathology, Faculty of Medicine and Health Sciences, United Arab Emirates University, Al-Ain, United Arab Emirates; 2 Departments of Paediatrics, Faculty of Medicine and Health Sciences, United Arab Emirates University, Al-Ain, United Arab Emirates; Cornell University, United States of America

## Abstract

Hereditary haemorrhagic telangiectasia (HHT) is an autosomal dominant genetic condition affecting the vascular system and is characterised by epistaxis, arteriovenous malformations and mucocutaneous and gastrointestinal telangiectases. This disorder affects approximately 1 in 8,000 people worldwide. Significant morbidity is associated with this condition in affected individuals, and anaemia can be a consequence of repeated haemorrhages from telangiectasia in the gut and nose. In the majority of the cases reported, the condition is caused by mutations in either *ACVRL1* or *endoglin* genes, which encode components of the TGF-beta signalling pathway. Numerous missense mutations in endoglin have been reported as causative defects for HHT but the exact underlying cellular mechanisms caused by these mutations have not been fully established despite data supporting a role for the endoplasmic reticulum (ER) quality control machinery. For this reason, we examined the subcellular trafficking of twenty-five endoglin disease-causing missense mutations. The mutant proteins were expressed in HeLa and HEK293 cell lines, and their subcellular localizations were established by confocal fluorescence microscopy alongside the analysis of their N-glycosylation profiles. ER quality control was found to be responsible in eight (L32R, V49F, C53R, V125D, A160D, P165L, I271N and A308D) out of eleven mutants located on the orphan extracellular domain in addition to two (C363Y and C382W) out of thirteen mutants in the Zona Pellucida (ZP) domain. In addition, a single intracellular domain missense mutant was examined and found to traffic predominantly to the plasma membrane. These findings support the notion of the involvement of the ER's quality control in the mechanism of a significant number, but not all, missense endoglin mutants found in HHT type 1 patients. Other mechanisms including loss of interactions with signalling partners as well as adverse effects on functional residues are likely to be the cause of the mutant proteins' loss of function.

## Introduction

Hereditary hemorrhagic telangiectasia or Osler-Rendu-Weber syndrome is a genetically heterogeneous autosomal dominant vascular disorder characterized by multiorgan vascular dysplasias, recurrent epistaxis and mucocutaneous telangiectasia [1]–[3]. Prevalence of HHT is estimated to be at least 1 in 8,000 with higher rates seen in some geographical areas [4]–[6].

Individuals with HHT initially present with spontaneous recurrent nosebleeds from telangiectasia of the nasal mucosa [2], [7], [8]. Telangiectases may also develop on the face, lips, mouth and gastrointestinal tract leading to haemorrhage and anemia in some cases [2], [8]. Unfortunately, arteriovenous malformations (AVMs) in the pulmonary, cerebral or hepatic circulation account for some of the most devastating clinical complications of HHT including stroke, fatal hemorrhages and heart failure [9].

HHT can be classified into at least two types; type 1 (HHT1; OMIM 187300) is caused by mutations in Endoglin (*ENG*) gene and type 2 (HHT2; OMIM 600376) is caused by mutations in activin receptor-like kinase 1 (*ACVRL1*) gene [4], [10], [11]. Mutations in both genes account for nearly 85% of all HHT cases while the remaining cases are caused by mutations in *SMAD4* or other yet unknown genes [11].

The protein products of *ENG* and *ACVRL1* genes are type 1 membrane proteins and are components of the transforming growth factor beta (TGF beta) receptor. They are involved in intracellular signaling with biological implications on the regulation of cellular proliferation, differentiation, migration and extracellular matrix formation [12], [13]. Alk-1, the protein product of *ACVRL1* is a type 1 membrane receptor and a partner for BMPR2 protein whereas endoglin is an accessory receptor protein to the signaling complex [12], [14]–[16].

**Figure 1:**
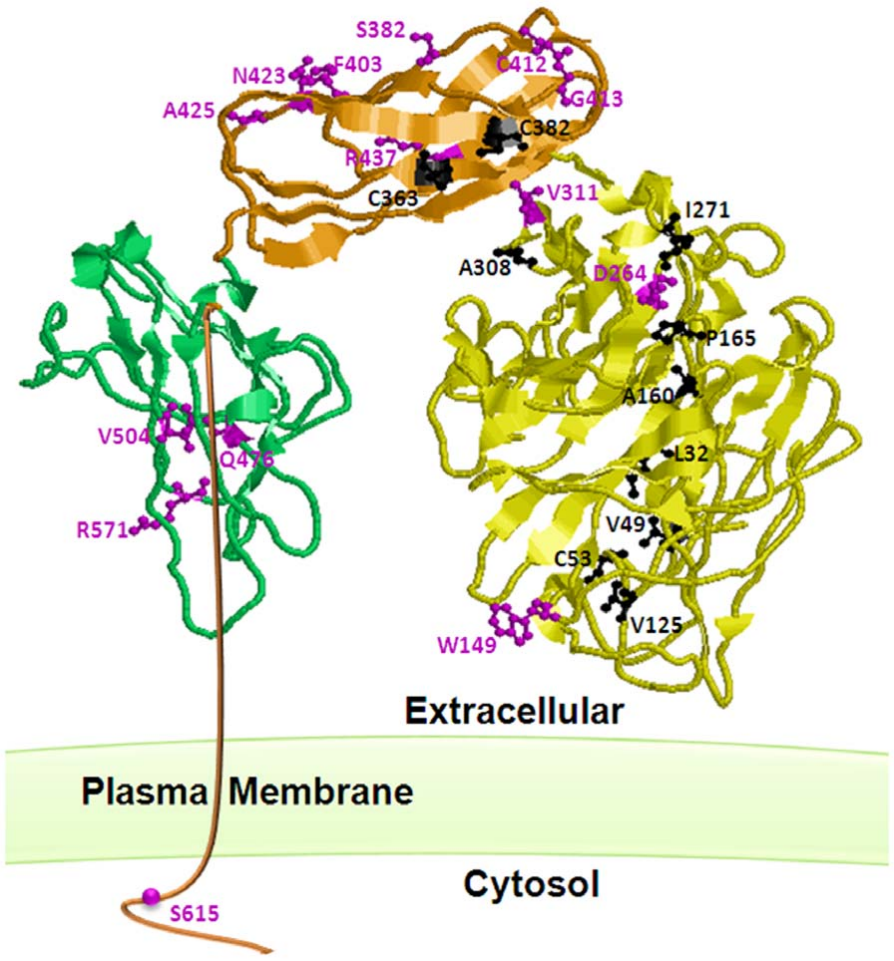
The three-dimensional structure of endoglin monomer showing the locations of the twenty five missense mutants studied in this aricle. Endoglin consists of a small c-terminal intracellular domain and three extracellular domains that include the ZP-C (green), ZP-N (Orange) and orphan (yellow) domains. The endoglin model structure (Llorca et al. 2007) file was provided by Dr Carmelo Bernabeu and then was manipulated using RasMol 2.7 (www.RasMol.org). The ball and stick represntation of the ER-retained (back) and the predominantly plasma membrane (purple) mutants are indicated on the strucure. It is clear that the majority of the mutants affecting the orphan domain resulted in the retention of the protein in the ER whereas those affecting the ZP domains retained their plasma membrane localization.

Over 700 different mutations in *ENG* and *ACVRL1* genes have been identified in patients with HHT1 and HHT2, respectively [4], [11], [17] (http://www.hhtmutation.org). Endoglin is a type I 180 KDa disulphide-linked homodimer integral membrane glycoprotein [13], [18], [19]. It contains a large extracellular domain of 561 amino acids that consists of Zona Pellucida (ZP) and orphan domains together forming a dome-like structure with an internal cavity in the dimeric state. In addition, it contains a small (47 amino acid) serine threonine rich intracellular domain of unknown function [19]. The cysteine residues in this protein are involved in intra- and inter-subunit disulfide bridges and this suggests a tightly folded and structured homodimer protein. The vast majority of HHT1 causing mutations in *ENG* are in the extracellular domain [4] (http://www.hhtmutation.org); this is presumably due to its much larger size compared to the intracellular domain (Fig. 1).

We hypothesized that many of the missense mutations affecting the disulfide bridges and other structural amino acids within the protein are expected to result in at least partial misfolding of the mutated proteins and subsequently their retention in the ER by the ER quality control mechanism. This eventually leads to degradation of these misfolded proteins by the ER-Associated protein Degradation (ERAD) system [20, B. Ali, unpublished].

ERAD is highly stringent and harbours an elaborate quality control mechanism for protein folding, posttranslational modifications and multisubunit complex assembly within the ER lumen of all eukaryotic cells [21]–[23]. Clearly, the ER is the entry point to the secretory pathway and therefore is the first step for almost a third of all cellular proteins. This means that several thousand different proteins are subjected to this highly stringent quality control system. We and others have shown that ERAD is involved in the cellular mechanisms of numerous human genetic conditions including cystic fibrosis, emphysema and Robinow syndrome [20], [24]–[27]. We exploited bioinformatics algorithms and databases of disease genes to identify over 30 ERAD disease candidates where a molecular defect consistent with protein misfolding was evident from the position and nature of the disease-associated allele, but significantly, where the molecular aetiology was unknown [20]. We experimentally validated this approach by the analysis of several autosomal recessive skeletal conditions including Robinow Syndrome [20], [27], Acromesomelic Dysplasia type Maroteaux [28] and Sponydylo-meta-epiphyseal Dysplasia with short limbs and abnormal calcifications (SMED-SL) [29]. In the vast majority of cases examined, disease-causing missense mutants were shown to be retained in the ER and degraded by ERAD. These findings provided unequivocal support to our bioinformatics predictive approach.

Unlike the conditions that we have examined previously, HHT1 is an autosomal dominant condition. However, it is considered to be a valid ER quality control candidate due to the loss-of-function nature and the haploinsufficiency effects of the causative mutations [30], [31]. In this article, we examine the trafficking of a significant number of missense mutations in endoglin that are found in HHT1 patients and demonstrate that retention in the ER by the ER quality control system significantly contributes to the cellular mechanism of this condition.

## Results

### Mutations in the orphan domain of endoglin are predominantly localized to the endoplasmic reticulum

Structural modelling of the extracellular domains of the human endoglin protein revealed two major domains, an orphan N-terminal domain that comprise residues Glu26-Ile359 and ZP domain that comprises residues Gln360-Gly586 [19]. The predicted orphan domain structure does not show significant homology to any known family of proteins. However, an excess of 150 different mutations within this domain, including many missense mutations, have been shown to cause HHT1 (http://www.hhtmutation.org). Indeed, 21 out the 33 missense mutations modelled by Llorca et al [19] are located in the orphan domain and the vast majority of them are conserved within different species. Reduced or absence of cell surface expression of some HHT1 causing mutants have been reported previously [4]. However, the underlying mechanism of this reduced expression and the overall mechanism of HHT1 have not been fully established. In addition, we previously hypothesized that the ER quality control might be contributing to the cellular mechanisms of HHT [20, B. Ali, unpublished].

**Figure 2:**
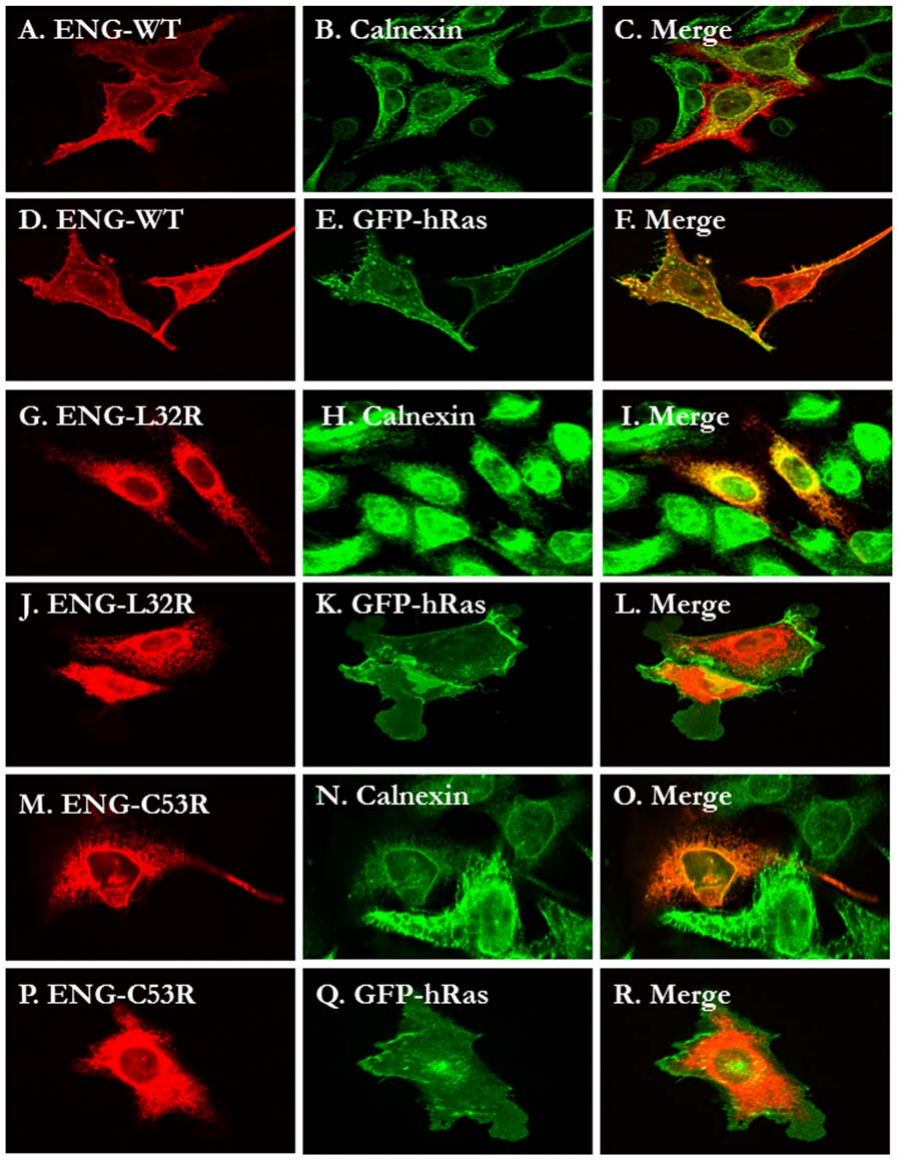
Comparison of the subcellular localization of wild type Endoglin with two (L32R and C53R) orphan domain HHT1-causing mutants. HeLa cells were transiently transfected with the C-terminally HA-tagged Endoglin pCMV5 plasmids (panels A-C, G-I and M-O) or co-transfected with the same plasmids and the EGFP-tagged H-Ras plasmid (Panels D–F, J–L and P–R) and processed for fluorescence confocal microscopy as described in the methods. The HA tagged proteins were detected with Anti-HA monoclonal antibodies (red panels A, D, G, J. M and P) and the ER marker calnexin was detected with anti-calnexin polyclonal antibodies (panels B, H and N). GFP-H-Ras staining is shown in panels E, K and Q. The wild type predominantly showed plasma membrane localization as evidenced by its co-localization with Ras (D–F). On the other hand the two mutants (L32R and C53R) showed ER localization (G–R).

**Figure 3:**
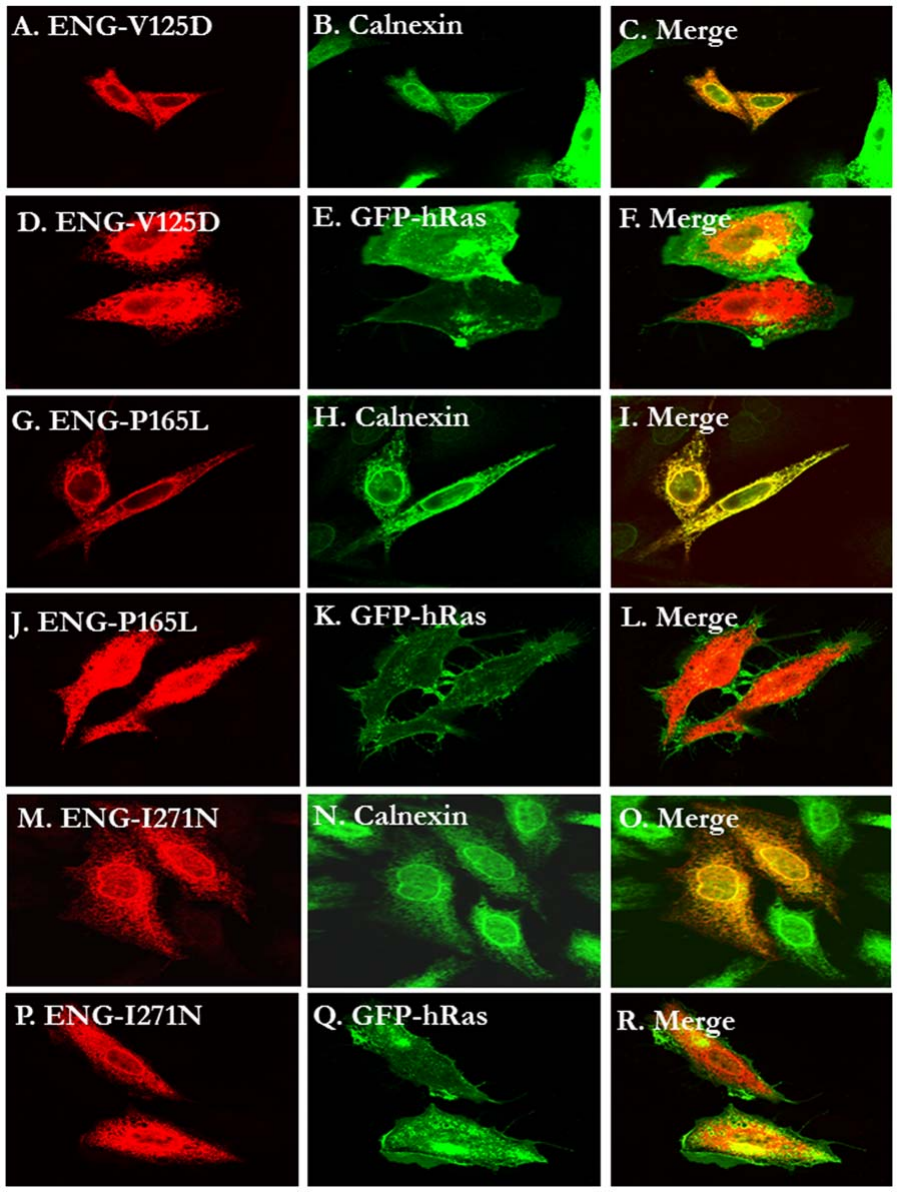
The subcellular localization of three orphan domain HHT1-causing mutants (V125D, P165L and I271N). HeLa cells were transiently transfected with the C-terminally HA-tagged Endoglin pCMV5 plasmids (panels A–C, G–I and M–O) or co-transfected with the same plasmids and the EGFP-tagged H-Ras plasmid (Panels D–F, J–L and P–R) and processed for fluorescence confocal microscopy as described in the methods. In all three cases, the mutants co-localized with the ER marker (A–C for V125D; G–I for P165L and panles M–O for I271N) but not to the plasma membrane (D–F, J–L and P–R for V125D, P165L and I271N, respectively).

Randomly selected missense mutants (L32R, V49F, C53R, V125D, W149C, A160D, P165L, D264N, I271N, A308D and V311G) within the orphan domain were generated by site directed mutagenesis. Their cellular localization by confocal fluorescence microscopy and sensitivity to endoglycosidase H (Endo H) treatment was also established. We show that eight of the eleven mutations resulted in the mislocalization of endoglin to the ER rather than to the plasma membrane. As shown in the confocal microscopy images in figure 2, the wild type endoglin is predominantly localized to the plasma membrane as evidenced by its co-localization with GFP-hRas. The partial localization with the ER marker (calnexin) is most likely indicative of the in transit newly synthesized endolglin population. Alternatively, many wild type secretory proteins that fail to fold properly are retained in the ER for some time to allow folding to take place. On the other hand, the missense mutants L32R, C53R, V125D, P165L and I271N are predominantly co-localized with calnexin and were absent from the plasma membrane (Fig. 2 and 3). In addition, the following mutants V49F, A160D and A308D co-localized with calnexin in the ER and were excluded from the plasma membrane (confocal microscopy images not shown). Only W149R, D264N and V311G mutants were found to be localized to the plasma membrane in a pattern reminiscent to the wild type endogin (data not shown).

To further confirm the subcellular localization of the generated mutants mentioned above, we performed Endo H sensitivity and resistance *in vitro* assays on the expressed proteins. Endoglin is a highly glycosylated protein with several potential N-glycosylation sites in its extracellular domain [18], [19]. The premise of this assay is that the carbohydrate moieties of the ER-localized glycoproteins are cleavable by Endo H whereas the post-ER species will be resistant due to further remodelling in the Golgi complex of their N-glycans.

**Figure 4:**
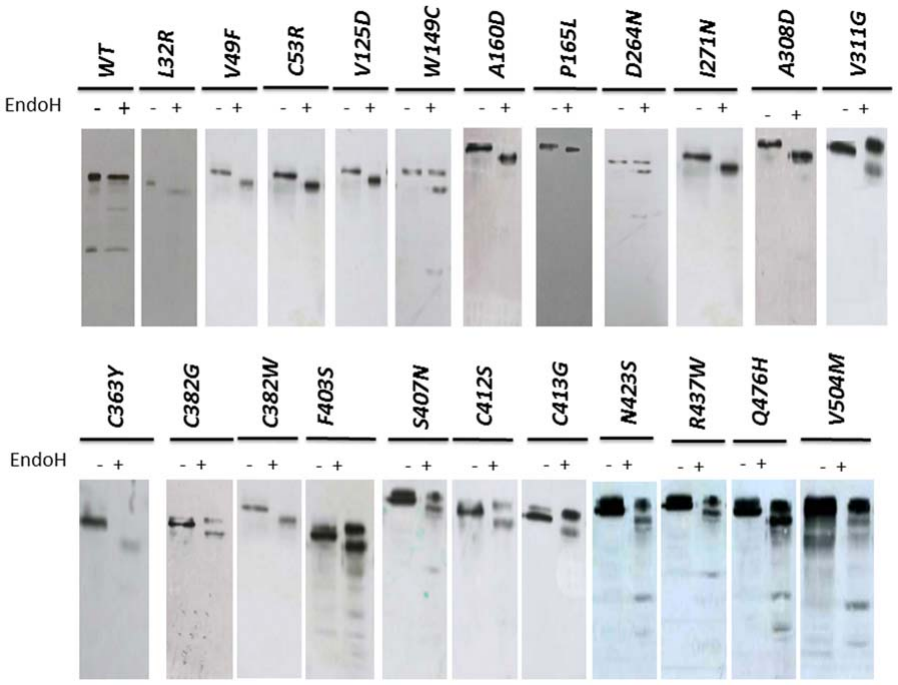
Endoglycosidase H (EndoH) analysis of the expressed missense mutants. The pCMV5 HA-tagged endoglin plasmids were transfected into HEK293 cells and were allowed to express the proteins as described in the methods. This was followed by lysis of the cells and the immuoprecipitation of the proteins with anti-HA monoclonal antibodies. Each immunoprecipitate was divided into two sample with one of them was treated (+) with EndoH or left untreated (−). Both samples were then electrophorese and western blotted using anti-HA monoclonal antibodies as described in the methods section. The majority of the WT protein did not change mobility upon EndoH treatment indicating its maturation and acquisition of complex N-glycan moieties in the post ER compartments. The missense mutants L32R, V49F, C53R, V125D, A160D, P165L, I271N, A308D, C363Y and C382W showed a single band indicating an exclusively ER premature forms. The other mutants either showed two bands or a simple high molecular weight band indicating that at least a significant proportion of the expressed protein matured in post ER compartments. The EndoH treatment data are in agreement with the confocal microscopy data.

We performed the Endo H assay on immuneprecipitated wild type and mutants proteins expressed in HEK293 cells as described in the method section. As expected, we found that a proportion of the wild type protein is resistant to Endo H treatment and confirms that the wild type protein targets largely to the plasma membrane (Fig. 4) as observed in figures 2 and 3. On the other hand, mutants L32R, V49F, C53R, V125D, A160D, P165L, I271N and A308D showed a single lower molecular weight protein band after Endo H treatment and thus confirming their retention in the ER and failure to pass through the Golgi complex (Fig. 4). Three mutants that appeared to localize predominantly to the plasma membrane (W149R, D264N and V311G) behaved in a similar manner to the wild type upon Endo H treatment. Summary of the confocal microscopy and Endo H assay data is summarized in table 1.

**Table 1:** Summary of the subcellular localization of the missense mutants described in this study.

Mutant	Domain	Experimental localization*
Wild type	None	Predominantly PM
L32R	Orphan Domain	ER
V49F	Orphan Domain	ER
C53R	Orphan Domain	ER
V125D	Orphan Domain	ER
W149C	Orphan Domain	Predominantly PM
A160D	Orphan Domain	ER
P165L	Orphan Domain	ER
D264N	Orphan Domain	Predominantly PM
I271N	Orphan Domain	ER
A308D	Orphan Domain	ER
V311G	Orphan Domain	Predominantly PM
C363Y	ZP-N Domain	ER
C382G	ZP-N Domain	Predominantly PM
C382W	ZP-N Domain	ER
F403S	ZP-N Domain	Predominantly PM
S407N	ZP-N Domain	Predominantly PM
C412S	ZP-N Domain	Predominantly PM
G413V	ZP-N Domain	Predominantly PM
N423S	ZP-N Domain	Predominantly PM
A425G	ZP-C Domain	Predominantly PM
R437W	ZP-N Domain	Predominantly PM
Q476H	ZP-C Domain	Predominantly PM
V504M	ZP-C Domain	Predominantly PM
R571H	ZP-C Domain	Predominantly PM
S615L	Intracellular Domain	Predominantly PM

*Subcellular localization have been established by confocal microscopy and/or Endo H analysis.

### Mutations in the ZP and the intracellular domains of endoglin are predominantly localized to the plasma membrane

The other domains of endoglin are the ZP extracellular domain, which can be divided into ZP-N and ZP-C sub-domains, the 25 hydrophobic residue transmembrane domain (Leu587-Trp611) and the intracellular domain [19]. The ZP-N domain comprises residues Gln360-Ser457 and ZP-C residues Pro458-Gly586. The intracellular cytoplasmic domain consist of 47 amino acids rich in serine and threonine residues of which three are constitutively phosphorylated [18], [32]. In a similar manner to the orphan domain, more than 150 different mutations, including many missense mutations, in the ZP domain have been identified in patients with HHT1. In contrast, only a couple of mutations have been reported within the small intracellular domain.

**Figure 5:**
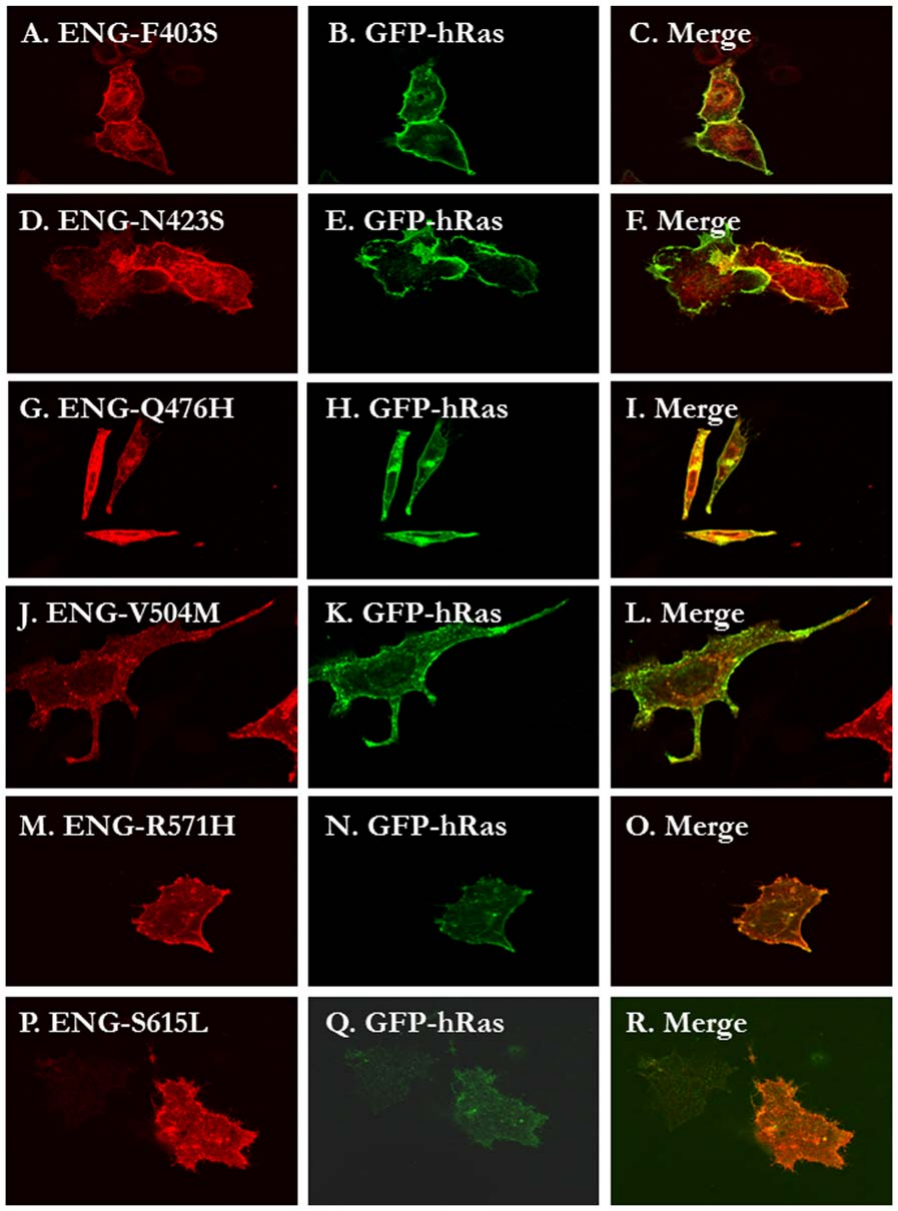
The majority of the missense mutants in the ZP and intraceullar domains traffic normally to the plasma membrane. HeLa cells were transiently co-transfected with the C-terminally HA-tagged Endoglin pCMV5 plasmids and the EGFP-tagged H-Ras plasmid and 24 hours later the cells were processed for fluorescence confocal microscopy as described in the methods. The following missense mutanst were shown to traffic predominantly to the plama membrane as eviednced by their co-localization with GFP-H-Ras: C382G ( data not shoiwn due to space limitations), F403S (panles A–C), S407N (not shown), C412S (not shown), G413V (not shown), N423S (panels D–F), R437W (not shown), A452G (not shown), Q476H (not shown), V504M (panels J–L), R571H (panels M–O ) and P615L (panels P–R).

We generated 13 missense mutations in the ZP domain and one mutation in the intracellular domain and we then evaluated the effects of these mutations on the intracellular localization of the mutated proteins, using either or both confocal microscopy and Endo H treatment analysis as described in the materials and methods section. The single intracellular missense mutation (S615L) had no effect on subcellular localization of the protein being predominantly localized to the plasma membrane in a similar pattern to wild type (data not shown). Similarly, the vast majority of ZP mutants examined (C382G, F403S, S407N, C412S, G413V, N423S, R437W, A452G, Q476H, V504M and R571H) behaved in a similar manner. Figure 5 illustrates representative examples of confocal images and figure 4 shows the Endo H treatment results. The only exceptions were C363Y and C382W mutants that co-localized with the ER marker (data not shown). The Endo H treatment results (Fig. 4) of these mutants confirmed the microscopy images.

## Discussion

HHT is an autosomal dominant condition affecting nearly 1 in 8,000 people worldwide. Complications associated with HHT can be serious, leading to significant morbidity and in some cases mortality. There is no cure for this condition and therefore there is an urgent need to search for effective treatments. Developing new avenues for therapy is likely to benefit from detailed understanding of the molecular and cellular basis of this condition. The main causes of HHT include mutations in *ENG*, the gene encoding endoglin which is an essential component of the TGF beta signaling machinery [33], [34].

In addition, haploinsufficiency has been proposed to be the most commonly accepted pathogenic model for HHT. Endoglin plays key roles in cardiovascular development, angiogenesis and vascular remodeling. Furthermore, it has been found that some missense endoglin mutations led to reduced or loss of cell surface expression of the mutant proteins [31], [35], [36]. However, the nature of the loss of cell surface expression and the universality of this mechanism to other missense mutations has not been established.

In this manuscript, we examined the subcellular trafficking and targeting of twenty five endoglin missense mutations found in HHT1 patients [4], [11], [17], [37] (http://www.hhtmutation.org). The examined missense mutations have been selected randomly and distributed along the protein structure including the small intracellular domain and the two large extracellular domains (the orphan and ZP domains). By means of electron microscopy and atomic modeling, Llorca et al [19] have determined a low resolution (25 Angstrom) three-dimensional structure of the extracellular domain of endoglin. They have found that the extracellular domain is arranged in a dome-like structure enclosing a cavity made of antiparallel oriented monomers each composed of three domains. The domain and structural locations of the studied missense mutations in this manuscript are shown in figure 1.

Subcellular localization of most of the missense mutations affecting the orphan domain resulted in the misfolding and presumably their retention by the ER quality control machinery (Fig. 2, 3, and 4). This domain has no structural similarities to other protein domains. However, from the *ab initio* low resolution three-dimensional model determined by Llorca et al [19], it is clear that this domain adopts a defined compacted structure with potential intramolecular disulphide bridges. Therefore, it is expected that structurally important missense mutations in this domain might lead to its improperly folded proteins, with subsequent retention in the ER, followed by ERAD.

As detailed in the introduction, ERAD is the main quality control mechanism operating at the entry point of the secretory pathways and therefore is responsible for ensuring the quality of almost a third of all the cellular proteins [20]–[23]. The first steps in ERAD are the recognition of the proteins as misfolded or malfolded followed by their retention within the ER lumen. In addition, this quality control machinery has been shown to be responsible for the mechanism of a large number of human genetic conditions [20], [24]–[26]. HHT is an ERAD disease candidate that has been identified using genetic disease databases and bioinformatics tools [20]. This was based on the nature of the missense mutations in ALK1- and endoglin that are causing HHT. In addition, it was clear from cellular studies that some of the missense mutants reduced or abolished the cell surface expression of the proteins [30], [31], [35], [38]. However, the retention within the ER and the involvement of ERAD has not been clearly established for these mutants.

Our data clearly implicate retention in the ER as a major mechanism of HHT1 missense mutants especially those harboring mutations in the orphan domain. The HHT1-causing missense mutants in endoglin that traffic to plasma membrane are likely to be causing the condition by different mechanisms including the loss of their ability to interact with partners or loss of their ability to activate the signaling pathways. It is also possible that the trafficking of those mutants is attenuated in comparison to the wild type protein thus compromising the protein biological function. However, we have no data at this stage to support the exact mechanisms of those mutants.

Elucidation of the mechanisms of disease is important for disease management and possibly developing new therapies. It has been recently proposed and shown in principle that manipulating ER-quality control and ERAD might be a feasible drug target for genetic diseases [39]–[46]. The realization, that some ER retained proteins might be biologically functional if they were given the opportunity to escape from this organelle has encouraged the search for manipulation of ERAD in ways to offer a therapeutic advantage in diseased states. This could be true for some of the endoglin missense mutants shown to be retained in the ER in the current study.

## Materials and Methods

### Chemicals and Reagents

Bovine serum albumin was obtained from Fisher Scientific (Loughborough, UK). Endoglycosidase H (Endo H) was purchased from New England Biolabs (Hertfordshire, UK). The antibodies and their sources were as follows: Antibodies for immunofluorescence: mouse anti-HA-Tag monoclonal antibody (dilution 1∶200; Cell Signaling Technology), rabbit anti-calnexin polyclonal antibody (dilution 1∶500; StressGen Biotechnologies), Alexa Fluor 568-goat anti-mouse IgG (dilution1∶200; Molecular Probes), Alexa Fluor 488-goat anti-rabbit IgG(dilution 1∶200; Molecular Probes). Secondary antibodies for Western blotting were as follows: rabbit-anti-goat Ig-horseradish peroxidase (Zymed Laboratories Inc., San Francisco, CA); sheep-anti-mouse Ig-horseradish peroxidase (Amersham Biosciences UK, Chalfont St Giles, UK).

### Construction of the HA-tagged Endoglin construct

The HA-tagged version of the wild-type endoglin was generated by sequential site-directed mutagenesis to introduce the nine amino acids of the HA tag (YPYDVPDYA) in two cycles using pCMV5-ENG as a template (a kind gift of Professor Michelle Letarte, University of Toronto, Canada). In the first cycle five amino acids (YPYDV) of the HA tag were introduced prior to the stop codon of the Endoglin gene using the following mutagenesis primers.

ENG-HA1-F: ccagcagcatggcatacccatacgatgtttagccccggccc

ENG-HA1-R: gggccggggctaagcgtaatctggaacatcgtatgggta

In the second cycle, the above construct was used as a template and the remaining four amino acids (PDYA) of the HA tag were introduced using the following mutagenesis primers.

ENG-HA2-F: tacccatacgatgttccagattacgcttagccccggccc

ENG-HA2-R: gggccggggctaagcgtaatctggaacatcgtatgggta

### Generation of missense Endoglin mutants

The missense mutants were introduced by QuickChange site-directed mutagenesis with Turbo *Pfu* polymerase (Stratagene) and using the C-terminally HA-tagged Endoglin as templates. The primers for the site-directed mutagenesis to introduce the missense mutants are as follows with the mutagenic nucleotide in bold and underlined:

ENG-L32R- F: 5′GTCCATTGTGACC**G**TCAGCCTGTGGG3′

ENG-L32R- F: 5′CCCACAGGCTGA**C**GGTCACAATGGAC3′

ENG-V49F-F: 5′CATATACCACTAGCCAG**T**TCTCGAAGGGCTGCGTG3′

ENG-V49F-R: 5′CACGCAGCCCTTCGAG**A**ACTGGCTAGTGGTATATG3′

ENG-C53R-F: 5′GGTCTCGAAGGGC**C**GCGTGGCTCAGGCCC3′

ENG-C53R-R: 5′GGGCCTGAGCCACGC**G**GCCCTTCGAGACC3′

ENG-V125D-F: 5′GTAAACAGCAGTG**A**CTTCCTGCATCTCC3′

ENG-V125D-R: 5′GGAGATGCAGGAAG**T**CACTGCTGTTTAC3′

ENG-W149C-F: 5′GATCCTTGAGTG**C**GCAGCTGAGAGG3′

ENG-W149C-R: 5′CCTCTCAGCTGC**G**CACTCAAGGATC3′

ENG-A160D-F: 5′CATCACCTCTGCTG**A**TGAGCTGAATGACC3′

ENG-A160D-R: 5′GGTCATTCAGCTCA**T**CAGCAGAGGTGATG3′

ENG-P165L-F: 5′GAGCTGAATGACC**T**CCAGAGCATCCTCC3′

ENG-P165L-R: 5′GGAGGATGCTCTGG**A**GGTCATTCAGCTC3′

ENG-D264N-F: 5′GTCCTGGCTCATC**A**ACGCCAACCACAAC3′

ENG-D264N-R: 5′GTTGTGGTTGGCGT**T**GATGAGCCAGGAC3′

ENG-I271N-F: 5′ CACAACATGCAGA**A**CTGGACCACTGGAG 3′

ENG-I271N-R: 5′CTCCAGTGGTCCAG**T**TCTGCATGTTGTG3′

ENG-A308D-F: 5′CCGGATGCTCAATG**A**CAGCATTGTGGCATC3′

ENG-A308D-R: 5′GATGCCACAATGCTG**T**CATTGAGCATCCGG3′

ENG-V311G-F: 5′CAATGCCAGCATTG**G**GGCATCCTTCGTG3′

ENG-V311G-R: 5′CACGAAGGATGCC**C**CAATGCTGGCATTG3′

ENG-C363Y-F: 5′GATCCAGACAAAGT**A**TGCCGACGACGCC3′

ENG-C363Y-R: 5′GGCGTCGTCGGCA**T**ACTTTGTCTGGATC3′

ENG-C382G-F: 5′GCGCATTTGAAG**G**GCACCATCACGG3′

ENG-C382G-R: 5′CCGTGATGGTGC**C**CTTCAAATGCGC3′

ENG-C382W-F: 5′CGCATTTGAAGTG**G**ACCATCACGGGC3′

ENG-C382W-R: 5′GCCCGTGATGGT**C**CACTTCAAATGCG3′

ENG-F403S-F: 5′GGGGTGACAAGT**C**TGTCTTGCGCAG3′

ENG-F403S-R: 5′CTGCGCAAGACA**G**ACTTGTCACCCC3′

ENG-S407N-F: 5′GTTTGTCTTGCGCA**A**TGCTTACTCCAGCTG3′

ENG-S407N-R: 5′CAGCTGGAGTAAGCA**T**TGCGCAAGACAAAC3′

ENG-C412S-F: 5′GCTTACTCCAGC**A**GTGGCATGCAGG3′

ENG-C412S-R: 5′CCTGCATGCCAC**T**GCTGGAGTAAGC3′

ENG-G413V-F: 5′TACTCCAGCTGTG**T**CATGCAGGTGTCAG3′

ENG-G413V-R: 5′CTGACACCTGCATG**A**CACAGCTGGAGTA3′

ENG-N423S-F: 5′GTATGATCAGCA**G**TGAGGCGGTGGTC3′

ENG-N423S-R: 5′GACCACCGCCTCA**C**TGCTGATCATA 3′

ENG-A425G-F: 5′GATCAGCAATGAGG**G**GGTGGTCAATATCC3′

ENG-A425G-R: 5′GGATATTGACCACC**C**CCTCATTGCTGATC3′

ENG-R437W-F: 5′GCTCATCACCACAG**T**GGAAAAAGGTGCAC3′

ENG-R437W-R: 5′GTGCACCTTTTTCC**A**CTGTGGTGATGAGC3′

ENG-Q476H-F: 5′GAGCTTTGTGCA**C**GTCAGAGTGTCCC3′

ENG-Q476H-R: 5′GGGACACTCTGAC**G**TGCACAAAGCTC3′

ENG-V504M-F: 5′GAGGGAGGCACC**A**TGGAACTCATCCAGG3′

ENG-V504M-R: 5′CCTGGATGAGTTCCA**T**GGTGCCTCCCTC3′

ENG-R571H-F: 5′GGACTGTCTTCATGC**A**CTTGAACATCATCAG3′

ENG-R571H-R: 5′CTGATGATGTTCAAG**T**GCATGAAGACAGTCC3′

ENG-S615L-F: 5′CTGGTACATCTACT**T**GCACACGCGTTCC3′

ENG-S615L-R: 5′GGAACGCGTGTGC**A**AGTAGATGTACCAG3′

All constructs were confirmed by direct DNA sequencing of the purified plasmids.

### DNA Sequencing

Sequencing was performed using the dideoxy method by fluorescent automated sequencing on the ABI 3130xl genetic analyzer (Applied Biosystems, Foster City, USA). The sequence data were analysed using Sequencing analysis software v5.3 (ABI, Foster City, USA).

### Cell culture and transfection

HeLa cells were cultured in Dulbecco's modified Eagle's medium (Invitrogen) supplemented with 10% fetal calf serum, 2 mM L-glutamine and 100 U/ml penicillin/streptomycin at 37°C with 10% CO_2_. For transfection, cells were grown on sterile cover slips in a 24 well tissue culture plates. Transfection was performed after 24 h using the liposomal transfection reagent FuGENE (HD Reagent, Promega, USA)according to manufacturer's instructions. For each well, 0.55 µg of the Endoglin wild type and the mutant constructs were used to transfect the cells. Twenty four hours after transfection, the cells were fixed and processed for microscopy.

Human embryonic kidney (HEK) 293 cells (ATCC, Manassas, VA) were cultured in Dulbecco's modified Eagle's medium/F12 medium (Invitrogen) supplemented with 10% fetal bovine serum(FBS) (Sigma Chemical Co, MO, USA), penicillin (10 units/ml) and streptomycin (100 µg/ml) at 37°C in a humidified atmosphere of 5% CO incubator. One day before transfection, cells were seeded on six multiwells (Ø35 mm) and grown to 70% to 80% confluency. Transfection was performed, using FuGENE HD Transfection Reagent (Promega, USA) according to the manufacturer's instructions. Cells cultured were transfected with 2 µg of Endoglin wild-type or mutant plasmid DNAs.

### Immunohistochemistry

For immunofluorescence, cover slip-grown HeLa cells were washed with phosphate-buffered saline (PBS), fixed by methanol at −20°C for 4 minutes. Fixed cells were then washed in PBS three times and incubated in blocking solution (1% BSA in PBS) for 30 min at room temperature. The fixed cells were then incubated for 1 h at room temperature with either mouse monoclonal anti-HA antibodies alone or co-incubated with rabbit polyclonal anti-calnexin antibodies. After washing with PBS, the cells were incubated with the appropriate fluorescently-labelled secondary antibodies for 1 h at room temperature then washed several times with PBS and mounted in immunofluor medium(ICN Biomedicals). We used EGFP-hRas as a marker for the plasma membrane. The H-Ras in this case was co-tansfected with the endoglin construct. Data were acquired using a Nikon confocal microscope 1(Japan). For presentation purposes, images were pseudo colored as either red (Endoglin) or green (calnexin and EGFP-H-Ras), contrast enhanced and overlayed using Adobe Photoshop® (Adobe Inc.). All images presented are single sections in the *z*-plane.

### Protein extraction and Immunoprecipitation

Immuoprecipitation was used to isolate the expressed Endoglin from cell lysates of HEK293 cells. Forty eight hours post-transfection of cell transfection, culture media was removed and cells were gently washed twice with ice cold PBS (Phosphate-buffered saline). After washing, 150 µl of 1× ice-cold lysis buffer (Cell-Lytic Mcell Sigma C2978 lysis reagent) containing protease inhibitors (SIGMAFAST™ Protease Inhibitor Cocktail Tablets, EDTA-Free; Sigma S8830) was added to the 3.5 cmPetri dishes and incubated on ice for 5 min. Cells were then scraped off the plate and transferred to microcentrifuge tubes and kept on ice for 15 min.The cell lysates were then centrifuged at 14000 g for 15 min at 4°C.The supernatants were then transferred to a new tube without disturbing the pellet. Protein was quantified by Pierce Bicinchoninic Acid protein Assay kit according to the manufacturer's instructions. Ten microliters of protein A was added to 100 µl of cell lysate and incubated on a rotator for 45 min at 4°C. After centrifugation at 2500 g for 3 min the supernatant was transferred to a fresh 1.5 ml tube taking care not to transfer any beads slurry. Then 1 µl of anti-HA antibodies were added to the supernatants and incubated at 4°C on a rotator for 3 h then 10 µl of protein A (pre equilibrated in the corresponding IP lysis buffer) was added and incubated at 4°C for 2 h to capture the immune complex. The tubes were centrifuged at 2500 g for 30 s at 4°C and the supernatant was carefully removed and the beads were washed once with 100 µl of cold lysis buffer.

### Endoglycosidase H Digestion

After immunoprecipitation, 10 µl of protein A-Sepharose beads containing the immune complex (Endoglin and anti HA antibody) was diluted by adding 10 µl of water. Then 5 µl of sodium phosphate buffer (250 mM, pH 5.5) and 1.25 µl of denaturation (containing 2%SDS and 1 M 2-mercaptpethanol, Sigma product number S4927) solution added to each sample. Samples were then denatured by heating at 100°C for 5 min. After cooling, 2 µl of Endoglycosidase H (Sigma, Saint Louis, USA) was added to each sample and incubated at 37°C for 3 h. The cleavage products were subjected to SDS-PAGE and western blotting using anti-HA antibodies as described below.

### Western blotting analysis

Thirty µg of protein from control (untransfected cell), endoglin wild type and mutant constructions were subjected to15% SDS-polyacrylamide gel and transferred tonitro cellulose membrane. After 1 h blocking in 5% BSA (Bovine Serum Albumin), nitrocellulose blots were incubated overnight with anti-HA antibody (1∶1000, Santa Cruz Biotechnology).Following washing with TRIS-buffered saline/Tween-20 (0.05%), membranes were incubated with goat anti-mouse IgGHRP (1∶50000, Santa Cruz Biotechnology)for 1 h and developed according to standard procedures.
